# Measles outbreak in a community with very low vaccine coverage, the Netherlands.

**DOI:** 10.3201/eid0707.010743

**Published:** 2001

**Authors:** S van den Hof, C M Meffre, M A Conyn-van Spaendonck, F Woonink, H E de Melker, R S van Binnendijk

**Affiliations:** National Institute of Public Health and the Environment, Bilthoven, The Netherlands. Susan.van.den.Hof@rivm.nl

## Abstract

A 1999-2000 measles epidemic in the Netherlands started with an outbreak in an orthodox reformed elementary school with 7% vaccine coverage. The overall attack rate was 37%: 213 clinical cases among the 255 participating pupils (response 62%) and 327 household members. The attack rate ranged from 0% for the oldest groups of pupils to 88% for the youngest, who had not been exposed in previous measles epidemics. None of 25 vaccinated pupils had clinical symptoms. Among pupils with clinical symptoms, the self-reported complication rate was 25%. These data confirm that measles infection causes severe disease and that vaccination is the most effective means of preventing the disease and its complications. The data also show that clusters of persons refraining from vaccination interfere with measles elimination even in populations with very high overall vaccine coverage (96%).


### Measles Outbreak in a Community with Very Low Vaccine Coverage, the Netherlands

**Susan van den Hof,* Christine M.A. Meffre,***
**Marina A.E. Conyn-van Spaendonck,* Frits Woonink,†**
**Hester E. de Melker,* and Rob S. van Binnendijk***
*National Institute of Public Health and the Environment, Bilthoven, the Netherlands;
and †Public Health Service Southeast Utrecht, Zeist, the Netherlands

A 1999-2000 measles epidemic in the Netherlands started with an outbreak in an orthodox reformed elementary school with 7% vaccine coverage. The overall attack rate was 37%: 213 clinical cases among the 255 participating pupils (response 62%) and 327 household members. The attack rate ranged from 0% for the oldest groups of pupils to 88% for the youngest, who had not been exposed in previous measles epidemics. None of 25 vaccinated pupils had clinical symptoms. Among pupils with clinical symptoms, the self-reported complication rate was 25%. These data confirm that measles infection causes severe disease and that vaccination is the most effective means of preventing the disease and its complications. The data also show that clusters of persons refraining from vaccination interfere with measles elimination even in populations with very high overall vaccine coverage (96%).

In the Netherlands, measles vaccination started in 1976, with 14-month-old babies. A two-dose schedule, implemented in 1987, offered a combined measles, mumps, and rubella (MMR) vaccine to 14-month-old babies and 9-year-old children. The national vaccine coverage for both doses of MMR is 96% (1), but this rate is not uniform throughout the country. In 1999, 34 (6%) of the 539 municipalities had vaccine coverage of <90% for the first dose of MMR (1). These 34 municipalities, which are concentrated in a geographic belt from the southwest to the mideast of the country, contain clusters of orthodox reformed communities, most of whose members refrain from vaccination on religious grounds. The communities (estimated population 300,000, 2% of Dutch population) form a strongly coherent social group that has its own churches and schools and consists of large families (2). Notification data show that measles epidemics have mainly affected unvaccinated persons and have occurred every 5 to 7 years since the introduction of vaccination: in 1976, 1983, 1987-1988, 1992-1994, and 1999-2000 (3,4).

The most recent epidemic was first noticed on June 21, 1999. Five cases of measles were reported to a Public Health Service (PHS) in the Netherlands by a general practitioner (GP) from a municipality with low vaccine coverage (78% for the first dose of MMR)(1,5). The five patients all attended the same regional, orthodox reformed elementary school. Two days later, the headmaster informed PHS that 80 (19%) of the 412 pupils were ill at home.

After laboratory confirmation (specific serum immunoglobulin [Ig] M antibodies) of the first clinical cases, we started a study with a twofold aim: 1) to evaluate alternative methods for diagnosing measles (including detection of specific IgM antibodies in saliva and measles virus in oropharyngeal swabs and urine through reverse transcriptase-polymerase chain reaction [RT-PCR]) and 2) (on which this article reports) to assess the attack rates among pupils and their families and the severity of disease associated with measles infection.

### Methods

### Study Population

We sought participation of all patients whose cases were reported to PHS between June 21 and July 2, 1999, and their household contacts, as well as all pupils from grade 1 (n = 48, 5 and 6 years of age) of the orthodox reformed elementary school. We requested two house calls, the first right before summer holidays (July 2), and the second right after the holidays (August 23). On the first visit, a questionnaire was completed and blood, saliva, oropharyngeal swab, and urine specimens were collected from all consenting household members, even those without symptoms. On the second visit, a questionnaire was completed, and blood and saliva specimens were obtained. On the first questionnaire, demographic variables, symptoms, and history of measles, measles vaccination status, travel abroad, and contact with measles were detailed. On the second visit, the section on symptoms was completed, if applicable, and two forms with additional questions were filled out. The first form inquired about complications, GP consultations, hospitalization, and medication. On the second form, limited information was gathered on all other household members (date of birth, sex, and recent measles infection).

Pupils from grades 2 to 8 were sent the same questionnaires and additional questions before and after summer vacation. We received the names of pupils in grade 0

Address for correspondence: Susan van den Hof, National Institute of Public Health and the Environment, Department of Infectious Diseases Epidemiology, P.O. Box 1, 3720 BA Bilthoven, the Netherlands; fax: 3130-274-4409; e-mail: Susan.van.den.Hof@rivm.nl

(preschool, which is voluntary) after vacation; therefore, we sent them the questionnaires in August only. Measles vaccination history of all 412 pupils of the school (grades 0 to 8) was verified at the Provincial Vaccination Administrations (PVA).

### Laboratory Tests

The presence of specific serum IgM antibodies was determined with a commercially available IgM-capture enzyme-linked immunosorbent assay (ELISA) according to procedures recommended by the manufacturer (Meddens mu-capture ELISA for measles, Biotest, Denville, NJ). IgG antibody concentrations were measured by an in-house ELISA (6).

### Case Classification

We classified cases according to a modified Centers for Disease Control and Prevention case definition (7): Confirmed cases had ≥3 days of rash, fever ≥38.3°C, and either cough, conjunctivitis, or coryza; suspected cases had rash and fever according to questionnaire or recent measles according to form, with limited information on household members of pupils. We considered positive serologic results (positive IgM or a minimal fourfold rise in IgG titer) or virus isolation from blood or oropharyngeal swab to be laboratory evidence of measles infection.

### Data Analyses

Attack rates for clinically confirmed and suspected measles cases were calculated by sex, year of birth, vaccination history, history of measles, and susceptibility, i.e., no vaccination, no history of measles, and birth in 1986 or later. Persons born before 1987 experienced measles epidemics in 1987-88 and in 1992-93. This was confirmed by the fact that we observed only one clinical case (the patient was born in 1986) among all persons born before 1987 (n = 226). Therefore, we considered all persons born before 1986 without information on history of measles to have had measles.

Vaccine efficacy estimates were based on the attack rate of measles among pupils who reported no history of measles in the questionnaire and by vaccination history as given by PVA. Symptoms and complications as reported in the questionnaires were described for those who had clinically confirmed or suspected measles and who had completed at least one questionnaire. We used the chi-square test to test differences in attack rates regarding categorical variables. A p value of <0.05 was considered statistically significant.

### Results

### Response

Responses to questionnaires, limited information, and collected biological samples (from pupils and household members) are shown in Table 1. All families with one or more reported measles patients from June 21 through July 2, 1999, had elementary school pupils in their households. We obtained questionnaires on 299 persons and limited information on 283 of their household members from 123 families, and we obtained biological samples from 100 persons in 26 families.

Table 1. Participation in measles outbreak investigation, the Netherlands, 1999–2000

| | Pupils (n = 412) | | Household members (n = 375)[a] | | Total (n = 787) | |
|---|---|---|---|---|---|---|
| Sources | n | % | n | % | n | % |
| Questionnaire and biological samples[b] | 50 | (12) | 36 | (10) | 86 | (11) |
| Questionnaire | 197 | (48) | 16 | ( 4) | 213 | (27) |
| Limited information and biological samples | 0 | ( 0) | 14 | ( 4) | 14 | ( 2) |
| Limited information (August only) | 8 | ( 2) | 261 | (70) | 269 | (34) |
| No information | 157 | (38) | 48 | (13) | 205 | (26) |

[a] Number of household members not attending the elementary school of the 255 participating pupils.
[b]Questionnaires for all 86 participants from whom questionnaires and biological sample(s) were collected both in July and August.

### Description of the Outbreak

In total, 213 cases of measles (110 confirmed and 103 suspected) were identified (Table 2); 138 were in pupils. All suspected cases were epidemiologically linked to a confirmed case through school or family contacts. Therefore, we consider suspected cases true measles cases and describe our results for the confirmed and suspected cases together.

The epidemic curve is shown in Figure 1. Day 1 of rash was known in 137 of the 213 confirmed and suspected cases and occurred from June 15 to July 20, 1999. The number of

Table 2. Measles cases by clinical and laboratory case classification, the Netherlands, 1999–2000

| Clinical case classification | Number | Number providing biological samples | Positive laboratory confirmation |
|---|---|---|---|
| Confirmed | 110 | 41 | 34[a] |
| Suspected | 103 | 10 | 5[a] |
| Noncase | 369 | 48 | 5[b] |
| Total | 582 | 99[c] | 44 |

[a]All 12 clinically confirmed and suspected cases without laboratory confirmation had no rash until 3-20 days after sampling.
[b]Details on these 5 persons are in Table 4.
[c]Here, 99 persons with laboratory case classification are shown instead of 100 as in Table 1, since 1 person did not provide blood and throat swab specimens, but only saliva and urine.

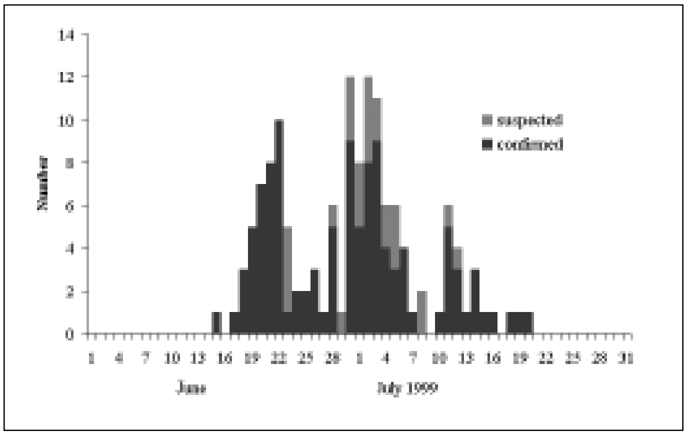

Figure 1. Distribution of clinically confirmed and suspected cases by date of onset of rash (n = 137).

persons per household was 3 to 18 (median 6). The number of reported cases per household was 0 to 9 (median 2): 37 (30%) households reported no cases, including 12 (10%) households with children vaccinated against measles.

### Attack Rates

The overall attack rate among confirmed and suspected cases was 37% (Table 3), 0% for the oldest pupils to 88% for the youngest (Figure 2). Two (1%) of the 213 patients were born in 1999; 166 (78%) from 1992 to 1998; and 43 (20%) from 1988 to 1991. Two (1%) patients were born before 1988 (1986 and 1987). The distribution of cases and attack rate by sex, vaccination history, history of measles, and susceptibility (i.e., no vaccination, no history of measles, and born in or after 1986), is shown in Table 3. Except for sex, all variables were associated with the attack rate ($p < 0.05$).

The attack rate among susceptible pupils was 91% (133 of 146). Of the 28 nonpupils considered susceptible, 24 (86%) had clinical cases (Table 3). Three of the four who did not become ill were probably protected by maternal antibodies (date of birth from December 1998 to April 1999).

Among the 69 pupils considered not susceptible because of reported history of measles, one had clinical symptoms and laboratory confirmation of measles infection (Table 3). According to the questionnaire, this grade 1 pupil had measles in 1998. No vaccination was registered at PVA. This child probably had another rash disease in 1998. No cases were observed among the 195 nonpupils considered not susceptible (Table 3).

### Laboratory Results

The diagnosis was laboratory confirmed for 39 of the 51 clinically confirmed and suspected cases with one or two biological samples, the first of which was collected at or just after Day 1 of rash (IgM postive or IgG titer rise). We had collected only one sample in each of the remaining 12 cases; measles rash did not develop in these patients until 3 to 20 days later. As expected, IgM antibodies could not be detected in these cases. Five of 48 asymptomatic persons who had provided biological samples had laboratory evidence of measles infection (Tables 2, 4).

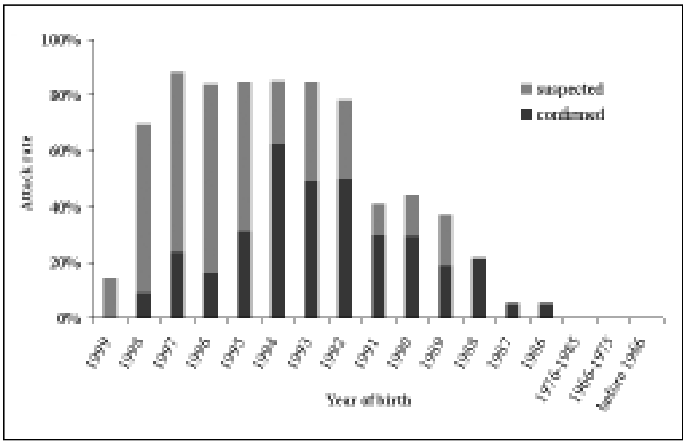

Figure 2. Attack rates by year of birth for clinically confirmed and suspected cases (n = 213).

### Vaccination History and Vaccine Efficacy

Of all 412 pupils, 28 (7%) had been vaccinated, according to PVA records. Of the 255 participating pupils, 25 (10%) had been vaccinated: 20 had one dose of MMR vaccine, and 5 had had two doses. None of the 25 vaccinated pupils reported measles symptoms (Table 3). Four (one parent and three young children) (10%) of the 42 nonpupils with a questionnaire reported vaccination against measles. None reported symptoms.

### Symptoms and Complications

The median number of days the rash lasted was 5 (10th-90th percentile, 3-9), the median number of days with fever was 6 (10th-90th percentile, 3-9). Of the 148 patients with confirmed or suspected measles who had given at least one answer about symptoms during measles disease, 54 (37%) reported Koplik's spots; 93 (63%) itching; 139 (94%) coughing; 136 (92%) conjunctivitis; 116 (78%) sore throat; 101 (68%) coryza; 86 (58%) diarrhea; 57 (39%) vomiting; 79 (53%) headache; and 28 (19%) aching joints.

Table 3. Attack rates (ARs) for clinically confirmed and suspected measles cases among pupils and their household contacts, by sex, vaccination history, history of measles, and susceptibility, the Netherlands, 1999–2000

| | Pupils | | | Household members | | | Total | | | |
|---|---|---|---|---|---|---|---|---|---|---|
| | n | Cases | AR (%) | n | Cases | AR (%) | n | (%) | Cases | AR (%) |
| Sex | | | | | | | | | | |
| Male | 129 | 69 | (53) | 174 | 38 | (22) | 303 | (52) | 107 | (35) |
| Female | 126 | 69 | (55) | 153 | 37 | (24) | 279 | (48) | 106 | (38) |
| Vaccination history[a] | | | | | | | | | | |
| Vaccinated | 25 | 0 | ( 0) | 4 | 0 | 0 | 29 | ( 5) | 0 | ( 0) |
| Unvaccinated | 230 | 138 | (62) | 48 | 24 | (50) | 278 | (48) | 162 | (58) |
| Unknown | 0 | 0 | 0 | 275 | 51 | (18) | 275 | (47) | 51 | (19) |
| History of measles | | | | | | | | | | |
| Yes | 69 | 1 | ( 2) | 192 | 0 | ( 0) | 261 | (54) | 1 | ( 0) |
| No | 168 | 133 | (79) | 39 | 26 | (67) | 207 | (36) | 159 | (77) |
| Unknown | 18 | 4 | (31) | 96 | 49 | (47) | 114 | (20) | 53 | (45) |
| Susceptibility[b] | | | | | | | | | | |
| Yes | 146 | 133 | (91) | 28 | 24 | (86) | 174 | (30) | 157 | (90) |
| No | 92 | 1 | ( 1) | 195 | 0 | ( 0) | 287 | (49) | 1 | ( 0) |
| Unknown | 17 | 4 | (31) | 104 | 51 | (49) | 121 | (21) | 54 | (45) |
| Total | 255 | 138 | (54) | 327 | 75 | (23) | 582 | (100) | 213 | (37) |

[a]Vaccination history for pupils according to Provincial Vaccine Administration records, for nonpupils according to questionnaire.
[b]Susceptibility of pupils: with no recorded measles-containing vaccination(s) and with no reported history of measles. Susceptibility of nonpupils: with no reported measles-containing vaccination(s) and with no reported history of measles for those born in 1986 or later.

Table 4. Asymptomatic persons with laboratory confirmation of measles virus infection, the Netherlands, 1999–2000

| Participant | Year of birth | History of measles | Measles vaccination | Laboratory confirmation |
|---|---|---|---|---|
| 1 | 1974 | Unknown | No | IgM positive<br>Data on IgG titer rise not available |
| 2 | 1980 | Yes | Data missing | IgM negative<br>≥fourfold IgG titer rise |
| 3 | 1982 | Yes | Data missing | IgM negative<br>≥fourfold IgG titer rise |
| 4 | 1992 | No | No | IgM negative<br>≥fourfold IgG titer rise |
| 5 | 1994 | No | Yes | IgM negative<br>≥fourfold IgG titer rise |

IgM = immunoglobulin M; IgG = immunoglobulin G.

Of the 162 patients with confirmed or suspected measles who completed at least one questionnaire, 40 (25%) reported one or more complications; one of the 40 was hospitalized for delirium (Table 5). Of the 40 patients with complications, 27 (68%) consulted GPs, who prescribed medication for 22 (55%) children. Of the 22 children, 19 were given antibiotics: 9 for pneumonia, 9 for otitis media, and 1 for cystitis. Antipyretic and analgesic medications were also prescribed. The complication rate did not differ between confirmed and suspected cases (26% vs. 24%).

Table 5. Self-reported complications in clinically confirmed and suspected cases, from questionnaire data, the Netherlands, 1999–2000

| Self-reported complications | Number | % |
|---|---|---|
| Hospitalization for delirium | 1 | 0.6 |
| Otitis media | 18 | 11 |
| Pneumonia | 10 | 6 |
| Earache | 5 | 3 |
| Stomachache | 3 | 2 |
| Cystitis | 1 | 0.6 |
| Laryngitis | 1 | 0.6 |
| Severe coughing | 1 | 0.6 |
| No complications | 113 | 70 |
| Data missing | 9 | 6 |
| Total | 162 | 100 |

### Conclusion

We have described an outbreak of measles in a mostly unvaccinated population. From this outbreak, measles spread and affected mainly (94%) unvaccinated persons from orthodox reformed communities. By May 2000, 3,292 cases of measles were reported to the national registry, including three measles-related deaths and 72 hospitalizations.

### Attack Rates

The susceptibility levels and attack rates were closely related to the number of previous epidemics encountered; those persons born after 1992, when the last epidemic began, had the highest susceptibility levels and attack rates. The 1999 birth cohort and part of the 1998 birth cohort are exceptions because they were partially protected by maternal antibodies. Sex was not associated with the attack rate, which is in accordance with previous reports (8). The infectivity of the measles virus is shown by the high attack rate (90%) among those considered susceptible (i.e., those with no history of measles or vaccination).

### Import and Export of Measles Virus

Measles viruses isolated from patients showed that the epidemic was caused by a D6 type measles virus, a genotype widely distributed throughout Europe (9). Genotype D6 had frequently been isolated from unrelated cases in the Netherlands between 1993 and 1999 (van Binnendijk et al., unpub. data). During this period, the number of measles cases reported in the Netherlands decreased to one of the lowest rates in Europe (<1 per million in 1998). However, because of low vaccine coverage in orthodox reformed communities, the number of susceptible persons increases. Consequently, measles epidemics still occur, despite high national vaccine coverage and population immunity (1,6). Previously, we showed that measles is not endemic in the Netherlands, not even in areas with low vaccine coverage (10). This was confirmed in this 1999-2000 epidemic; no more cases were reported within 1 year after the start of the outbreak. Therefore, we assume that the epidemic was initiated by import from another country. Until the measles virus is eradicated, circulation will continue worldwide and epidemics will occur. During this epidemic, visiting relatives exported measles to Canada. The outbreak was restricted to 17 cases within an orthodox reformed community in Canada as a result of stringent measures (e.g., closing the school) (11).

### Laboratory Results

We observed five asymptomatic persons with serologic proof of measles infection. All had been in close contact with one or more measles patients. Two were children, one vaccinated (#5 in Table 4) and one without recorded measles vaccination or history of measles disease (#4). Incomplete immunity in the presence of residual maternal antibodies may have developed in the latter child during the 1992 measles epidemic (12). Two adults (#2 and #3) reported history of measles, the third (#1) reported no history of measles but might have had measles, on the basis of the year of birth. However, this person might also have had subclinical primary infection.

We assume that the increase in specific IgG (#2-#5) reflects secondary immune response in persons reexposed to measles virus, as has been demonstrated (13-15). We have not been able to detect virus, either by virus culture or RT-PCR from blood or oropharyngeal swab (data not shown), from any of these subclinically reinfected persons, as was recently shown for an immune mother of an adult measles patient (16). However, even if virus can be detected in blood, urine, or saliva, the critical issue is whether the virus load in these subclinically reinfected persons is high enough to transmit the measles virus.

### Vaccination History and Vaccine Efficacy

We observed low vaccine coverage (7% to 10%), but excellent vaccine effectiveness (100%) for the measles component of the MMR vaccine; none of the vaccinated persons had measles symptoms. In the measles epidemic following this outbreak, 5% of the reported cases patients were vaccinated; almost all of them had received one dose (5).

The real percentage of vaccinated patients is probably smaller. We expect that more vaccinated than unvaccinated persons with measles symptoms are seen and reported by GPs.

### Symptoms and Complications

Measles is sometimes thought of as a mild disease. However, we observed a self-reported complication rate of 25% for all patients, 68% of whom consulted a GP. We do not know whether children who did not complete a form on complications consulted a GP. The percentage of consultations for uncomplicated measles cases could be smaller than that for complicated cases. Therefore, the percentage of consultations for all cases may be overestimated.

The complication rate of 25% is based on self-reported complications, and the diagnosis was not always confirmed by a physician. This could explain why the complication rate is somewhat higher than expected for measles (8,12). Still, burden of disease was very high in the participating measles patients. During the following epidemic (1999-2000), three measles-related deaths and 72 hospitalizations were reported (5).

In this descriptive study of a measles outbreak with an attack rate of 90% among susceptible persons, we have shown that measles disease is severe, even in an industrialized country. Vaccination is the most effective means of preventing the disease and its complications. The national vaccine coverage of 96% for both doses of MMR is theoretically high enough to eliminate measles (17). However, despite this very high coverage, measles epidemics still occur as a result of areas with low vaccine coverage. In these sociodemographically clustered, mainly unvaccinated communities, the number of susceptible people increases, and consequently epidemics occur periodically. The clustering of unvaccinated persons is the critical factor for measles elimination in the Netherlands.

### Acknowledgments

We thank A. Dortú, T. Landwehr, and S. Hahné for their help in collecting the data; and E. van Boxtel, G. Berbers, P. van Gageldonk, and T. Marzec for technical support.

Dr. van den Hof is an epidemiologist in the National Institute of Public Health and the Environment, working with vaccine-preventable diseases.

### References

1. Vaccinatietoestand Nederland per 1 januari 1999. The Hague, The Netherlands: Inspectorate of Health; 2000.
2. Geubbels ELPE, Conyn-van Spaendonck MAE, Loon AM van. Poliomyelitis vaccinatie in Nederland. In: Gunning-Schepers LJ, Jansen J, editors. Volksgezondheid Toekomst Verkenning 1997. IV. Effecten van preventie. Maarssen: Elsevier/De Tijdstroom; 1997. p. 79-87.
3. Bijkerk H, Bilkert-Mooiman MAJ, Houtters HJ. De inentingstoestand bij aangegeven patiënten met mazelen tijdens de epidemie 1987/88. Ned Tijdschr Geneeskd 1989;133:29-32.
4. Hof S van den, Conyn-van Spaendonck MAE, Melker HE de, Geubbels ELPE, Suijkerbuijk AWM, Talsma E, et al. The effects of vaccination, the incidence of the target diseases. RIVM report no. 213676008. Bilthoven, The Netherlands: RIVM; 1998.
5. Measles outbreak--Netherlands, April 1999-January 2000. MMWR Morb Mortal Wkly Rep 2000;49:299-303.
6. Hof S van den, Berbers GAM, Melker HE de, Conyn-van Spaendonck MAE. Sero-epidemiology of measles antibodies in the Netherlands, a cross-sectional study in a national sample and in communities with low vaccine coverage. Vaccine 2000;18:931-40.
7. Measles, mumps, and rubella—vaccine use and strategies for elimination of measles, rubella, and congenital rubella syndrome and control of mumps: recommendations of the Advisory Committee on Immunization Practices (ACIP). MMWR Morb Mortal Wkly Rep 1998;47:1-57.
8. Black FL. Measles. In: Evans AS, editor. Viral infections of humans: epidemiology and control, 3rd ed. New York/London: Plenum Medical Book Company; 1989. p. 451-69.
9. Santibanez S, Heider A, Gerike E, Agafonov A, Schreier E. Genotyping of measles virus isolates from Central Europe and Russia. J Med Virol 1999;58:313-20.
10. Wallinga J, Hof S van den. Epidemiologie van mazelen in Nederland: een verkennende analyse van aangiften. Ned Tijdschr Geneeskd 2000;144:171-4.
11. Berichten van de LCI; mazelenbestrijding in Canada. Reported by Ruijs H. Infectieziekten Bulletin 2000;11:14.
12. Redd SC, Markowitz LE, Katz SL. Measles vaccine. In: Plotkin SA, Mortimer Jr EA, editors. Vaccines, 5th ed. Philadelphia: WB Saunders; 2000. p. 222-66.
13. Gustafson TL, Lievens AW, Brunnel PA, Moellenberg RG, Buttery CMG, Schulster LM. Measles outbreak in a fully immunized secondary-school population. N Engl J Med 1987;316:771-4.
14. Helfand FR, Kim DK, Gary HE, Edwards GL, Bisson GP, Papiana MJ, et al. Nonclassic measles infections in an immune population exposed to measles during a college bus trip. J Med Virol 1998;56:337-41.
15. Huiss S, Damien B, Schneider F, Muller CP. Characteristics of asymptomatic secondary immune responses to measles virus in late convalescent donors. Clin Exp Immunol 1997;109:416-20.
16. Vardas E, Kreis S. Isolation of measles virus from a naturally immune, asymptomatically re-infected individual. J Clin Virol 1999;13:173-9.
17. World Health Organization. Strategic plan for the elimination of measles in the European Region. CMDS 01 01 06/10. Copenhagen: WHO Regional Office for Europe; 1997.
